# Genetic variants associated with clinical characteristics and atopies in eosinophilic esophagitis

**DOI:** 10.1016/j.jacig.2026.100745

**Published:** 2026-06-04

**Authors:** Leticia Rodríguez-Alcolado, Marcos Navares-Gómez, Sergio Casabona-Francés, Javier Molina-Infante, Danila Guagnozzi, Paula Soria-Chacartegui, Sara Feo-Ortega, Laura Arias-González, Macarena Torres-Larrubia, Ronald Llerena-Castro, Verónica Martín-Domínguez, Jose Zamorano, Francisca Molina-Jiménez, Pedro L. Majano, Jennifer Fernández-Pacheco, Dolores Rivas, Elena Grueso-Navarro, Pablo Zubiaur, Miriam Saiz-Rodríguez, Rocío Juárez-Tosina, Francisco Abad-Santos, Cecilio Santander, Ángel Arias, Alfredo J. Lucendo, Emilio J. Laserna-Mendieta

**Affiliations:** a Department of Surgery, Medical and Social Sciences, Universidad de Alcalá, Alcalá de Henares, Madrid, Spain; b Department of Gastroenterology, Hospital General de Tomelloso, Tomelloso, Spain; c Centro de Investigación Biomédica en Red Enfermedades Hepáticas y Digestivas (CIBERehd), Madrid, Spain; d Department of Clinical Pharmacology, Hospital Universitario de La Princesa, Madrid, Spain; e Instituto de Investigación Sanitaria de La Princesa, Madrid, Spain; f Department of Gastroenterology, Hospital Universitario de La Princesa, Madrid, Spain; g Department of Gastroenterology, Hospital Universitario de Cáceres, Cáceres, Spain; h Department of Gastroenterology, Hospital Universitario Vall d’Hebron, Barcelona, Spain; i Department of Pediatrics, Hospital General de Tomelloso, Tomelloso, Spain; j Research Unit, Hospital San Pedro de Alcántara, Cáceres, Spain; k Research Unit, Fundación Burgos por la Investigación de la Salud (FBIS), Hospital Universitario de Burgos, Burgos, Spain; l Facultad de Ciencias de la Salud, Universidad de Burgos, Burgos, Spain; m Department of Pathology, Hospital General Mancha Centro, Alcázar de San Juan, Spain; n Faculty of Medicine, Universidad Autónoma de Madrid, Madrid, Spain; o Faculty of Medicine, Universidad Complutense de Madrid, Madrid, Spain; p Research Unit, Hospital General Mancha Centro, Alcázar de San Juan, Spain; q University Center for Health Sciences–HM Hospital (CUHMED), Camilo José Cela University and HM Hospital Health Research Institute, Madrid, Spain

**Keywords:** Eosinophilic esophagitis, genetic risk factors, stenosis, single nucleotide polymorphism, phenotype

## Abstract

**Background:**

Eosinophilic esophagitis (EoE) and atopy are associated with several genetic variants; however, their relationship with clinical features has been scarcely investigated.

**Objective:**

We sought to characterize the distribution of such variants and evaluate the association with endoscopic and histologic presentation of EoE and concomitant atopies.

**Methods:**

Thirty-eight single nucleotide polymorphisms (SNPs), previously linked to EoE risk, were analyzed in a large cohort of Spanish patients. An observational study was performed in patients with EoE recruited at 4 sites. SNP genotyping was performed using the OpenArray platform (Thermo Fisher Scientific) from DNA samples extracted from peripheral blood. To enhance the robustness and reliability of the identified genetic associations, patients were divided into discovery (70%) and validation (30%) cohorts.

**Results:**

Overall, 628 patients with EoE (74% male; mean age, 32 ± 15 years) were recruited. An association between EoE and SNPs located at the 5q22 locus was confirmed in our cohort; in addition, 2 new SNPs not previously associated with EoE in which the alternative allele distributed differently were identified. A multivariate model identified 4 SNPs (*ABCB1* rs1128503, *KCNJ2* rs312691, *STAT6* rs841718, and *TGFB1* rs8179181) predicting a mixed/stricturing phenotype. *CAPN14* rs74732520-CG/GG and rs77569859-TC/CC genotypes were associated with lower peak eosinophil counts at diagnosis. Regarding concomitant atopies, *IL13* rs1800925-TT was associated with increased risk for asthma and conjunctivitis, whereas *ABCB1* rs2032582-TT was linked to a reduced risk of dermatitis.

**Conclusions:**

The analysis of genetic variants in a Spanish population with EoE showed novel associations between specific SNPs and clinical characteristics of this disease.

## Graphical abstract

**Figure:**
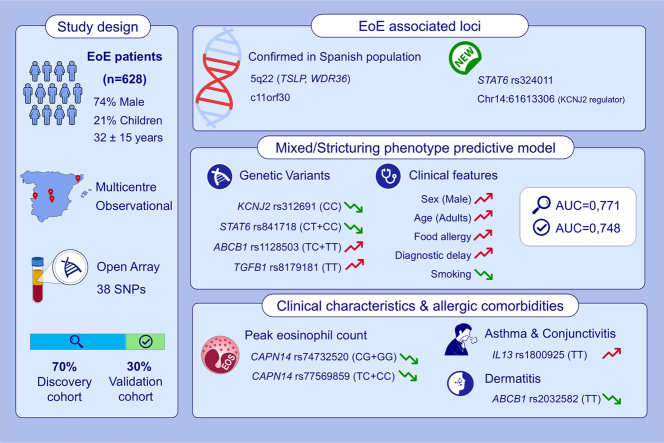

Eosinophilic esophagitis (EoE) is a distinct clinicopathological disorder, with a rapidly growing prevalence^1^^,^^2^ that leads to significant health care costs.^3^^,^^4^ EoE is mainly caused by food antigens that trigger a local type 2 inflammatory response.^5^ This leads to an eosinophil-predominant infiltration restricted to the esophagus and varying symptoms of esophageal dysfunction according to patients’ age and sex.^6^^,^^7^

To diagnose EoE, a peak eosinophil count greater than or equal to 15 cells/hpf (eos/hpf) in esophageal biopsies is required.^8^ In some patients, EoE may progress to fibrous remodeling, which aggravates symptoms, reduces effectiveness of therapy,^9^ and deteriorates patients’ quality of life.^10^ EoE phenotypes can be classified as inflammatory, mixed, or stricturing.^11^^,^^12^

Concomitant atopic conditions are commonly observed in patients with EoE, including a higher prevalence of bronchial asthma, conjunctivitis, dermatitis, and rhinitis, often accompanied by IgE-mediated food allergies.^13^^,^^14^ Common risk factors^15^^,^^16^ and shared pathophysiological mechanisms^17^^,^^18^ have been found in EoE and other atopic disorders.

Although environmental factors play a major role in the development of EoE,^19^^,^^20^ certain genetic variants predispose individuals to the disease.^21^ To date, approximately 70 single nucleotide polymorphisms (SNPs) have been associated with EoE, primarily located at 4 loci: 5q22 (*TSLP/WDR36*), 2p23 (*CAPN14*), 11q13 (*LRRC32/C11orf30*), and 12q13 (*STAT6*).^22^^,^^23^ Moreover, EoE has been classified into distinct endotypes on the basis of transcriptomic profiles derived from esophageal biopsies,^24^, ^25^, ^26^ which appear to distinguish important disease aspects. Furthermore, some risk SNPs affect genes whose dysregulation characterizes specific endotypes (eg, *TSLP*, *WDR36*, *CLECL16A*, and *TGFB1*),^23^^,^^24^^,^^26^ and a recent study has identified 32 EoE-associated variants that might regulate gene expression.^27^

However, the relationship between SNPs and the clinical characteristics of EoE, or the presence of concomitant atopic conditions, has not yet been fully explored. This study aimed to evaluate associations between SNPs linked to EoE and other atopic conditions and clinical features at diagnosis—including phenotype according to EREFS (exudates, rings, edema, furrows, and strictures) score and tissue eosinophilia—along with the presence of concomitant atopic conditions in a large multicenter cohort of Spanish patients with EoE. In addition, we compared the allele frequencies of SNPs previously associated with EoE risk with those observed in overall European and Iberian populations.

## Methods

### Study population

This is an observational study of patients diagnosed with EoE according to current guidelines^8^ at 4 Spanish hospitals (Hospital General de Tomelloso, Hospital Universitario de La Princesa, Hospital Universitario de Cáceres, and Hospital Universitario Vall d’Hebrón). The study was approved by the Research Ethics Committee of Hospital General Mancha Centro (internal code 185-C) and authorized by the Research Ethics Committees at the other participating hospitals.

Patients were recruited from March 2020 to July 2024. Patients newly diagnosed with EoE, or treated in gastroenterology clinics, or who underwent upper endoscopy as part of routine clinical management were invited to participate. No healthy subjects were recruited for this study.

After providing informed consent, a whole venous blood sample was obtained and frozen until DNA purification. To minimize potential sources of bias, standardized diagnostic criteria and uniform clinical data collection protocols were applied across all participating centers, following the EoE CONNECT standardized instructions.^12^ Clinical evaluators were blinded to genotyping results to reduce measurement bias. Given the relatively homogeneous European ancestry of the cohort, no formal adjustment was performed for population stratification.

Demographic and clinical data were obtained prospectively for patients with a new diagnosis, and for those in follow-up, data were extracted from the EoE CONNECT registry^12^ or retrieved retrospectively whenever possible. Clinical data recorded included ancestry, country of birth, EoE familiar background (in the study cohort), EoE phenotype, EREFS score at diagnostic endoscopy, peak of eos/hpf (defined as the highest peak among biopsies from any esophageal location), concomitant atopies (rhinitis, conjunctivitis, asthma, and dermatitis), and IgE-mediated food allergies. EoE phenotypes were defined on the basis of baseline endoscopic findings. A stricturing phenotype was defined by the presence of strictures or a narrow-caliber esophagus, alongside signs of inflammatory activity. Patients with a preserved esophageal caliber and an absence of rings or strictures were classified as having an inflammatory phenotype. Finally, the mixed phenotype was applied to cases with grade 2 concentric rings that did not impede the passage of the endoscope.^12^ All these data correspond to the time of diagnosis, before the initiation of any treatment for EoE.

The sample size was considered adequate to explore potential genetic associations on the basis of previous studies in similar populations and practical recruitment constraints. To increase the robustness, reproducibility, and reliability of the associations with genetic variants, the recruited cohort was randomly divided *a priori* into discovery (70%) and validation (30%) subcohorts.

### Genotyping

DNA extraction was performed using the Maxwell RSC Whole Blood DNA kit (Promega, Madison, Wis) in a Maxwell RSC instrument (Promega) following the manufacturer’s instructions.

Genotyping was performed using the QuantStudio 12K Flex Real-Time PCR System (Applied Biosystems, Thermo Fisher Scientific, Waltham, Mass) equipped with an OpenArray block and a custom-designed OpenArray plate (Thermo Fisher Scientific). The panel included 38 SNPs, among others, with selection based on (1) their biological or clinical relevance to EoE pathophysiology, (2) the level of functional evidence supporting their impact in the disease, and (3) the allelic frequency in the target population (mainly European, with small representation of African and Latin American ancestries) (see Table E1 in this article’s Online Repository at www.jaci-global.org). For this purpose, variant data were curated from peer-reviewed publications and public databases such as dbSNP, GWAS Catalog, and Ensembl.

Reaction mixes were prepared using TaqMan OpenArray Genotyping Master Mix (Thermo Fisher Scientific) and dispensed into the plates using the AccuFill system. PCR amplification was carried out following the manufacturer’s protocol. Genotype calling and clustering analysis were performed using the Thermo Fisher Cloud platform, which provides automated allele calling and quality control tools. To prevent potential bias during genotype assignment, genotyping was carried out blinded to the patients’ clinical characteristics.

A sample concordance analysis was conducted to identify potential duplicate or highly concordant samples. Given the limited number of SNPs genotyped (n = 60), this approach was restricted to detecting identical or first-degree related samples, because reliable inference of more distant relationships requires substantially higher marker density (>1000 SNPs). Although not a formal cryptic relatedness analysis, this method provides a robust assessment of genotype-level concordance. Genotypes were subjected to quality control, excluding SNPs with more than 5% missing values and samples with more than 10% missing data. Pairwise sample similarity was then assessed using the Pearson correlation coefficient as a global measure of genotype concordance. Sample pairs with correlation values greater than or equal to 0.98 were classified as highly similar, indicative of potential duplicates or sample misidentification.

The frequencies obtained for each genotype and allele are provided in Table E1. The less frequent alleles in our cohort were considered as variants to define dominant or recessive models (last column in Table E1). Gene consequences of the SNPs analyzed and known functional effects are provided in Table E2 (in the Online Repository available at www.jaci-global.org).

### Statistical analysis

Qualitative variables were described using absolute frequencies and percentages, whereas quantitative variables were presented as mean ± SD or as median with interquartile range. A chi-square test or the Fisher exact test was used to analyze qualitative variables, and those with a *P* value less than <.2 were checked for further grouping on following dominant or recessive models and subsequently retested. For the comparison of EREFS and peak of eos/hpf, the Kruskal-Wallis test was used, and those variables with a *P* value less than .1 were checked for further grouping following dominant or recessive models and subsequently tested with the Student *t* test or the Mann-Whitney *U* test, depending on whether the assumption of normality was met.

A multivariate model with binary logistic regression was used to identify potential independent risk factors for mixed/stricturing phenotypes at the time of EoE diagnosis in the discovery cohort. Clinical variables or SNPs that were significant, or showed a trend toward significance in univariate comparative analyses, were included in the model. Specifically, SNPs with a *P* value less than .2 in the univariate analysis, after grouping in dominant or recessive models, were considered as potential candidates. Odds ratios and their 95% CIs were calculated. Finally, the area under the receiver-operating characteristic curve (AUC) of the final model was calculated for both the discovery and validation cohorts.

Analyses were performed on available data. No imputation for missing values was conducted, and denominators are reported in the respective result tables. All analyses were carried out using PASW version 18.0 statistical analysis software (SPSS, Inc, Chicago, Ill) and GraphPad Prism version 5.0 (GraphPad Software, San Diego, Calif). Statistical significance was considered when *P* values were less than .05, and a statistical trend was considered when *P* values were between .05 and .2 in the validation cohort. Reporting of this study followed the STREGA (STrengthening the REporting of Genetic Association Studies) guidelines.^28^

## Results

### Characteristics of the cohorts

Overall, 628 patients with EoE were recruited. Table I provides demographic and clinical characteristics of the cohort, mainly young (mean age, 32.4 ± 15.2 years) male (74.0%) patients with an inflammatory phenotype (82%) at diagnostic endoscopy; 14% of them collected prospectively. As for atopies, rhinitis was the most common (55%), whereas dermatitis was the least frequent (17%). Food allergies were documented in 28% of patients. Family relationships were rare in our cohort, with 93% of patients having no relatives within it. Our patients had predominantly European ancestry, because only 10 individuals (1.6%) did not. In addition, 97.6% of the patients were born in Spain, further contributing to a relatively homogeneous genetic background in our cohort (see Table E3 in this article’s Online Repository at www.jaci-global.org).

**Table I:** Characteristics of the study cohort

Characteristics	Value
Type of data, n (%)	
Retrospective	539 (85.8)
Prospective	89 (14.2)
Hospital, n (%)	
Tomelloso	266 (42.4)
La Princesa	171 (27.2)
Cáceres	113 (18.0)
Vall d’Hebrón	78 (12.4)
Sex: male, n (%)	465 (74.0)
Age (y), mean ± SD	32.4 ± 15.2
Age group at diagnosis, n (%)	
<18 y	132 (21.0)
≥18 y	495 (78.8)
Unknown	1 (0.2)
EREFS	
Mean ± SD	3.14 ± 1.65
Median (IQR)	3 (2)
Peak of eos/hpf	
Mean ± SD	62.8 ± 45.6
Median (IQR)	50 (46)
Phenotype, n (%)	
Inflammatory	513 (81.7)
Mixed	35 (5.6)
Stricturing	79 (12.6)
Unknown	1 (0.2)
Presence of disease, n (%)	
Rhinitis	348 (55.4)
Conjunctivitis	242 (38.5)
Asthma	225 (35.8)
Dermatitis	109 (17.4)
Food allergies	177 (28.2)
At least 1 atopy, n (%)	462 (73.6)
Smoking status, n (%)	
Smoker	56 (8.9)
Former smoker	60 (9.6)
Never smoker	496 (79.0)
Unknown	16 (2.5)
Familiar relationships within the cohort, n (%)	
No	583 (92.8)
Parent-child	22 (3.5)
Siblings	14 (2.2)
Cousins	4 (0.6)
Second cousins	2 (0.3)
Multiple∗	3 (0.5)

*IQR*, Interquartile range.

∗ Multiple relationships include participants whose relatives belong to more than 1 category.

The characteristics of patients after allocation into the discovery (n = 439) or the validation (n = 189) cohort are provided in Table E4 (in the Online Repository available at www.jaci-global.org). No major differences were found between the 2 cohorts.

### SNPs associated with EoE in our cohort

We included 15 SNPs previously associated with increased or decreased risks for EoE. Among them, 10 variant alleles presented significantly different distributions in patients with EoE compared with the European population (Table II), whereas no differences were found for the other 5 SNPs (see Table E5 in this article’s Online Repository at www.jaci-global.org). In addition, 6 of these 10 displayed differences when the comparison was made against the Iberian population, thus indicating that many SNPs previously associated with EoE risk were also present in the Spanish population, including rs55646091 (c11orf30), and several SNPs in the 5q22 locus at *TSLP* and *WDR36* genes (rs3806932, rs1898671, rs2289276, rs1438673, and rs7723819).

**Table II:** SNPs with different allele frequencies in our patients with EoE (full cohort) compared with the European population

Gene	SNP (n, per group)	Major allele frequency (%)			Minor allele frequency (%)			*P* value (C vs EP)	*P* value (C vs IP∗)	SNP previously associated with EoE	References for SNPs previously associated with EoE
		EP	IP	C	EP	IP	C				
*ABCB1*	rs2032582 EP = 309909; IP = 214; C = 628	55.1	61.2	60.7	44.8 (T) 0.1 (A)	36.4 (T) 2.3 (A)	37.7 (T) 1.5 (A)	**<.001**	.620	No	—
*ABCG2*	rs2231142 EP = 311852; IP = 214; C = 627	89.7	93.0	94.0	10.3	7.0	6.0	**<.001**	.618	No	—
*ATP4A*	rs2733743 EP = 207628; IP = 214; C = 623	89.8	89.3	92.4	10.2	10.7	7.6	**.041**	.166	No	—
*c11orf30*	rs55646091 EP = 207628; IP = 214; C = 623	95.4	96.7	90.0	4.6	3.3	10.0	**<.001**	**.002**	Yes	Sleiman et al^29^
*CAPN14*	rs74732520 EP = 13116; IP = 214; C=623	97.6	94.4	92.5	2.4	5.6	7.5	**<.001**	.308	Yes	Sleiman et al^29^
chr14:61613308	rs2253681 EP = 20364; IP = 214; C = 613	87.5	89.7	83.8	12.5	10.3	16.2	**.005**	**.033**	No	—
*KCNJ2*	rs321691 EP = 20364; IP = 214; C = 613	75.4	72.9	71.7	24.6	27.1	28.3	**.029**	.732	No	—
*STAT6*	rs324015 EP = 258908; IP = 214; C = 616	76.2	73.8	81.1	23.8	26.2	18.9	**.005**	**.009**	No	—
*TGFB1*	rs1800469 EP = 499504; IP = 214; C = 623	68.4	61.7	63.2	31.5	38.3	36.8	**.004**	.703	Yes	Rawson et al^30^
*TGFB1*	rs1800470 EP = 13932; IP = 214; C = 616	52.5	57.9	57.1	47.5	42.1	42.9	**.026**	.819	No†	—
*TLR3*	rs3775292 EP = 14286; IP = 214; C = 623	80.6	73.8	75.5	19.4	26.2	24.5	**.002**	.630	Yes	Ávila et al^31^
*TSLP*	rs3806932 EP = 587068; IP = 214; C = 617	55.3	55.1	69.0	44.7	44.9	31.0	**<.001**	**<.001**	Yes	Rothenberg et al^32^
*TSLP*	rs10062929 EP = 79452; IP = 214; C = 624	86.0	90.7	91.7	14.0	9.3	8.3	**<.001**	.648	Yes	Sherrill et al^33^
*TSLP*	rs1898671 EP = 191656; IP = 214; C = 625	65.7	60.7	51.3	34.3	39.3	48.7	**<.001**	**.017**	Yes	Sherrill et al^33^
*TSLP*	rs2289276 EP = 209114; IP = 214; C = 626	70.4	65.4	78.0	29.6	34.6	22.0	**<.001**	**<.001**	Yes	Sherrill et al^33^
*TSLP/WDR36*	rs1438673 EP = 176420; IP = 214; C = 599	49.7	53.3	64.6	50.3	46.7	35.4	**<.001**	**.003**	Yes	Kottyan et al^34^
*WDR36*	rs7723819 EP = 410006; IP = 214; C = 625	55.5	55.6	68.6	45.5	44.4	31.4	**<.001**	**<.001**	Yes	Rothenberg et al^32^

*P* values were obtained using the χ^2^ test (or the Fisher exact test when appropriate). Significant *P* values were boldfaced.

*C*, Our cohort of patients with EoE; *EP*, European population; *IP*, Iberian population.

∗ Allele frequencies were also compared with those reported for the Iberian population.

† In linkage disequilibrium with rs1800469 in the European population (*P* < .001).

Other SNPs also distributed significantly differently within our patients with EoE compared with the European and Iberian populations (Table II). For example, despite being previously associated with EoE, rs324011 in *STAT6* was not differentially represented in our cohort; another SNP in the same gene, rs324015, had a lower proportion of the variant allele in our patients with EoE. Another SNP, rs2253681 in chr14:61613308, which is associated with asthma risk by modifying *KCNJ2* expression, also displayed different proportions of the alternative allele.

### SNPs and clinical variables associated with mixed/stricturing phenotype

We assessed the association between SNPs and clinical variables with the mixed/stricturing phenotype to develop multivariate models. The final model was selected on the basis of AUC values in both the discovery and validation cohorts. It included 5 clinical variables (sex, age at diagnosis, food allergies, smoking status, and diagnostic delay) and 4 genetic variants (Table III). Three of the clinical variables—sex, food allergies, and diagnostic delay—provided the greatest risks in the model.

**Table III:** Multivariate association by binary logistic regression for clinical variables and SNPs with mixed/stricturing phenotype (with inflammatory phenotype being the comparator group) at the moment of EoE diagnosis∗

Characteristics	OR (95% CI)	*P* value
Age group at diagnosis (adults vs children)	2.61 (1.10-6.20)	.029
Sex (male vs female)	3.91 (1.64-9.30)	.002
Food allergies (yes vs no)	3.13 (1.69-5.80)	<.001
Smoking (yes/former smoker vs no)	0.53 (0.24-1.16)	.113
Diagnostic delay (y)	1.09 (1.05-1.14)	<.001
*ABCB1* rs1128503 (DM, TC/TT vs CC)	1.74 (0.92-3.28)	.086
*KCNJ2* rs312691 (RM, CC vs TC/CC)	0.60 (0.31-1.14)	.119
*STAT6* rs841718 (DM, CC/CT vs TT)	0.49 (0.27-0.86)	.016
*TGFB1* rs8179181 (RM, TT vs CT/CC)	2.67 (0.86-8.32)	.089

*DM*, Dominant model; *OR*, odds ratio; *RM*, recessive model.

∗ The model was developed using 379 patients from the discovery cohort.

Two SNPs were found to protect against a mixed/stricturing phenotype: *KCNJ2* rs312691 (recessive model, present in 8% of patients and 10% of those with a mixed/stricturing phenotype) and *STAT6* rs841718 (dominant model, present in 61% of patients and 16% of those with the phenotype). In contrast, *TGFB1* rs8179181 was identified as a risk factor for the mixed/stricturing phenotype (recessive model, present in 6% of patients and 23% of those with the phenotype) and also *ABCB1* rs1128503 (dominant model, present in 65% of patients and 19% of those with the phenotype) (see Table E6 in this article’s Online Repository at www.jaci-global.org).

This model provided an AUC of 0.771 (95% CI, 0.710-0.833) for predicting the presence of a mixed/stricturing phenotype in the discovery cohort and performed similarly in the validation cohort, with an AUC of 0.748 (95% CI, 0.652-0.844). This model improved on the predictive performance demonstrated by the model proposed in our previous work on the influence of sex on the characteristics of EoE^7^ (which included sex, age group, smoking status, and diagnostic delay), as well as the model that incorporated food allergies, with this improvement being more evident in the validation cohort (Table IV).

**Table IV:** Comparison of the performance of the different multivariate models to predict the presence of mixed/stricturing phenotype at the moment of EoE diagnosis

Model	AUC DC	DC, n	AUC VC	VC, n
SexEoE CONNECT study∗	0.706 (0.641-0.772)	387	0.695 (0.598-0.791)	169
SexEoE CONNECT study + food allergies	0.754 (0.690-0.819)	387	0.706 (0.608-0.804)	169
SexEoE CONNECT study + food allergies + genetic variants	0.771 (0.710-0.833)	379	0.748 (0.652-0.844)	161

*AUC*, Area under the receiver-operating characteristic curve; *DC*, discovery cohort; *VC*, validation cohort.

∗ SexEoE CONNECT study refers to the multivariate model proposed in the study by Laserna-Mendieta et al,^7^ which included the following 4 clinical variables: age group at diagnosis, sex, diagnostic delay, and smoking status.

### SNPs associated with clinical characteristics of EoE

Two genetic variants were associated with endoscopic findings scored by EREFS at baseline endoscopy in the discovery cohort. However, we found no association for any of them in the validation cohort (see Table E7 in this article’s Online Repository at www.jaci-global.org).

Regarding the peak of eos/hpf in baseline esophageal biopsies, 6 genetic variants showed significant changes in the discovery cohort (Table E7), but only 2 SNPs (*CAPN14* rs74732520 and rs77569859) displayed a trend in the validation cohort (Table V). The presence of the alternative alleles (CG and GG genotypes in rs74732520, 14.4% of our patients; TC and CC genotypes in rs77569859, 23.1% of our patients) was associated with a reduced peak eosinophil count in esophageal biopsies. Both SNPs were in linkage disequilibrium in the European population (*r*^2^ = 0.470; *P* < .001).

**Table V:** Statistical analysis for association between SNPs in *CAPN14* gene with peak eosinophil count in each of the cohorts

SNP	Cohort	Genotype	n (%)	Mean ± SD	Range	*P* value
*CAPN14* rs74732520	Discovery cohort	CC	326 (84.9)	66.1 ± 48.3	15-500	.037
		CG, GG	58 (15.1)	55.3 ± 39.3	18-210	
	Validation cohort	CC	134 (83.2)	61.1 ± 43.9	16-300	.074
		CG, GG	27 (16.8)	47.8 ± 29.7	15-156	
*CAPN14* rs77569859	Discovery cohort	TT	343 (89.6)	66.6 ± 48.7	15-500	<.001
		TC, CC	40 (10.4)	47.6 ± 25.4	18-130	
	Validation cohort	TT	144 (89.4)	60.9 ± 43.6	15-300	.074
		TC∗	17 (10.6)	41.7 ± 18.9	23-80	

*P* values were calculated using the Student *t* test. Both SNPs are in linkage disequilibrium in the European population (*P* < .001).

∗ There were no homozygous subjects for the minor allele (GG genotype) in the validation cohort.

### SNPs associated with concomitant atopies and food allergies

We next evaluated the association of SNPs with rhinitis, conjunctivitis, asthma, dermatitis, and food allergies (Table VI; see also Table E8 in this article’s Online Repository at www.jaci-global.org). An association between *IL13* rs1800925 (TT genotype) and the presence of conjunctivitis was found (*P* = .029), which was also close to significance in the validation cohort (*P* = .056). Patients with EoE carrying this genotype (5.7% of the cohort) showed conjunctivitis in 60% of the cases, whereas this atopy was present in 39% of the overall cohort. This SNP was previously related to increased risk for asthma, but we exclusively found a trend for this association in the discovery cohort (*P* = .088), with similar distribution in the validation cohort. Asthma was present in 35% of patients with CC and CT genotypes in *IL13* rs1800925 and in 52% of those with the TT genotype. In addition, because an association of *STAT6* rs324015 with asthma was described for patients with EoE, we also investigated it in our cohort. A weaker association than that for *IL13* rs1800925 was found, because GA and AA genotypes (33.9% of the cohort) showed a trend (*P* = .061) with a lower odds ratio. Similar distribution was found in both the discovery and validation cohorts, and asthma increased from about 32% to 41% when the alternative allele was present.

**Table VI:** Statistical analysis for association between SNPs and atopic conditions in each of the cohorts

Variable	Gene and SNP	Cohort	Genotype	n (%)	Presence of AC (%)	OR (95% CI)	*P* value
Conjunctivitis	*IL13* rs1800925	Discovery cohort	CC, CT	413 (94.3)	38.0	2.4 (1.1-5.6)	.029
			TT	25 (5.7)	60.0		
		Validation cohort	CC, CT	177 (94.1)	35.0	3.2 (0.9-11.5)	.056
			TT	11 (5.9)	63.6		
Asthma	*IL13* rs1800925	Discovery cohort	CC, CT	413 (94.3)	35.1	2.0 (0.9-4.5)	.088
			TT	25 (5.7)	52.0		
		Validation cohort	CC, CT	177 (94.1)	33.3	2.4 (0.7-8.2)	.151
			TT	11 (5.9)	54.5		
	*STAT6* rs324015	Discovery cohort	GG	285 (66.1)	32.6	1.5 (1.0-2.2)	.061
			GA, AA	146 (33.9)	41.8		
		Validation cohort	GG	116 (62.7)	31.9	1.5 (0.8-2.7)	.232
			GA, AA	69 (37.3)	40.6		
Dermatitis	*ABCB1* rs2032582	Discovery cohort	GG, GT	365 (85.1)	18.4	0.3 (0.1-0.8)	.016
			TT	64 (14.9)	6.3		
		Validation cohort	GG, GT	154 (85.1)	20.8	0.5 (0.1-1.7)	.241
			TT	27 (14.9)	11.1		

*AC*, Atopic condition; *OR*, odds ratio.

Regarding dermatitis, this was found associated with *ABCB1* rs2032582. The presence in homozygosis of the alternative allele (TT genotype, 14.9% of our patients) protected against dermatitis (discovery cohort, *P* = .016; validation cohort, *P* = .241). The prevalence of this concomitance decreased from 18% to 6% in patients without or with this genotype, respectively, in the discovery cohort.

Finally, no associations were found in the discovery cohort for rhinitis and food allergies. Other SNPs associated with asthma and dermatitis were not confirmed in the validation cohort (Table E8).

## Discussion

In this study, we analyzed a Spanish cohort of patients with EoE to assess the frequency and impact of SNPs previously associated with risk for EoE or atopy on the clinical characteristics of the disease as well as their association with concomitant atopic diseases. To our knowledge, this is the first study to address the relationship between genetic variations and clinical characteristics of EoE in a large European cohort, comprising nearly 630 individuals with EoE, recruited at 4 different regions of Spain.

Of the 15 SNPs previously linked to EoE risk included in our analysis, significant differences in allele frequencies regarding the general European population were found in 10,^35^ and 6 of these also differed significantly from the Iberian subpopulation, according to the 1000 Genomes Project.^36^ In addition, we observed differences in allele frequencies in 2 SNPs not previously linked to EoE risk, with 1 previously associated with asthma (rs2253681 on chromosome 14), which likely reflects the connection between EoE and other concomitant atopies.^37^^,^^38^

We developed a multivariate model to examine the association of genetic and clinical variables with the presence of a mixed/stricturing phenotype, typically associated with a more complex disease course and reduced treatment efficacy.^39^, ^40^, ^41^, ^42^ Our results are consistent with findings from our previous large-scale European study of nearly 3000 patients from the EoE CONNECT registry,^7^ which identified male sex, adult age at diagnosis, and diagnostic delay as risk factors for a mixed/stricturing phenotype, whereas smoking was protective. Notably, our study also identified food allergies as an additional risk factor. This aligns with previous research suggesting that food allergies may predispose patients to a more treatment-resistant form of EoE.^43^^,^^44^

Regarding genetic variants, we identified 2 SNPs (*ABCB1* rs1128503-TC/TT and *TGFB1* rs8179181-TT) associated with an increased risk of developing the mixed/stricturing phenotype, whereas 2 others (*KCNJ2* rs312691-CC and *STAT6* rs841718-CC/CT) exhibited a protective effect. None of these SNPs are known to have functional or clinical consequences, because *ABCB1* rs1128503 is a synonymous variant and the remaining 3 are intronic. Although little is known about them, these variants likely exert some regulatory effects. For instance, variant allele in *ABCB1* rs1128503 can alter mRNA stability, which may modify the levels of the protein (P-glycoprotein).^45^ The intronic *STAT6* rs841718 was proposed as a cis–expression quantitative trait locus, modifying *STAT6* expression,^46^ which in turn could modulate T_H_2-mediated eosinophilic pathways. Similarly, the variant allele of *KCNJ2* rs312691 could reduce Kir2.1 expression, potentially affecting the channel function.^47^ In addition, *KCNJ2* has been previously linked to EoE risk, because it is overexpressed in esophageal biopsies^26^ and oral mucosa^48^ of patients with active disease; however, how rs312691 affects the EoE phenotype remains unclear. Nevertheless, SNPs individually exhibited modest effect sizes on risk when compared with clinical variables, and environmental factors, such as tobacco exposure and diagnostic delay, also act as significant contributors.^49^^,^^50^

The multivariate model demonstrated consistent discriminatory performance in both the derivation and validation cohorts, with AUC values of 0.77 and 0.75, respectively. Although these values fall short of the 0.8 threshold typically considered indicative of a strong predictive model, and therefore it is not yet suitable for implementation in routine clinical practice, they highlight the potential utility of incorporating genetic data into predictive frameworks to improve phenotypic classification and risk stratification.

We found 2 SNPs in *CAPN14* associated with peak of eos/hpf at diagnosis. The presence of the variant alleles, CG/GG genotypes for rs74732520 and CC/CT for rs77569859, was linked with a lower peak of eos/hpf. This finding further supports the involvement of *CAPN14* in the pathogenesis of EoE, consistent with its specific expression in the esophagus and its regulatory role in maintaining epithelial barrier integrity.^51^

We additionally identified other associations between SNPs and concomitant atopic conditions. *IL13* rs1800925 TT genotype was associated with a higher prevalence of conjunctivitis and showed a trend toward increased asthma rates. This regulatory variant enhances *IL13* promoter activity in human T_H_2 lymphocytes, thereby increasing the risk of allergic disorders.^52^ Other variants within the *IL13* gene have been linked to the risk of rhinoconjunctivitis.^53^

Compared with a previous study that explored the associations between SNPs and baseline characteristics of EoE in a small cohort of 28 patients,^54^ we also observed a trend toward an association between asthma and GA and AA genotypes in *STAT6* rs324015. Patients carrying the alternative allele had a 1.5-fold increased likelihood of having asthma. In contrast, we could not replicate previously reported associations between *STAT6* rs12368672 and food allergies or EREFS score, according to our analysis of rs324011, which is in linkage disequilibrium with rs12368672. The association between rs324015 and asthma has shown inconsistent results across studies; a meta-analysis found a significant effect only on atopic asthma in the subgroup analyses.^55^ Interestingly, the alternative allele of rs324015 was less prevalent in our EoE cohort compared with the general European and Iberian populations.

Furthermore, we found that the TT genotype of *ABCB1* rs2032582 (also known as G2677T) protected against dermatitis. Although *ABCB1* is well known for its role in drug transport, no previous studies have established a direct association between this SNP and atopic dermatitis. However, evidence from murine models suggests that P-glycoprotein may protect against pruritus and inflammatory responses in atopic dermatitis, and its expression is reduced in the skin of affected patients.^56^^,^^57^ These findings align with our results, because the TT genotype is associated with increased *ABCB1* expression.^58^

Our study has several notable strengths, including the use of a large and well-characterized Spanish cohort of patients with EoE from 4 different geographic areas and the integration of clinical and genetic data. The inclusion of a validation cohort further enhances the reliability and robustness of our findings.

Nevertheless, certain limitations should be acknowledged. First, the sample size may have been underpowered to identify relationships involving less common variants (with minor allele frequency <5%). Second, this study did not include some SNPs recently associated with EoE, such as those located in risk loci of the *CLEC16A* (16q13), *RAD50* (5q31), *RORA* (15q22), *SMAD3* (15q23), and *JAK2* (9p24) genes.^27^^,^^59^, ^60^, ^61^ Third, we did not perform formal multiple-testing correction (eg, Bonferroni); instead, we used a 2-stage design with independent discovery and validation cohorts to mitigate false-positive findings through replication. Therefore, the absence of formal multiple-testing correction could increase the risk of false-positive findings, and thus results should be interpreted as exploratory. Finally, the technology used did not allow for the identification of novel associations through genome-wide genotyping arrays based on high-throughput sequencing technologies. Therefore, although our findings provide meaningful insights into the association between SNPs and clinical characteristics of EoE, they should be confirmed in future analyses involving larger and more diverse populations and using a broader set of SNPs.

In conclusion, our study identified novel associations between specific SNPs that confer risk for EoE and atopic conditions as well as genetic variations associated with an increased risk of mixed/stricturing phenotypes. Further validation of these genetic associations, along with a deeper molecular understanding of their functional roles, may ultimately lead to the identification of predictive biomarkers that could help define the disease course and guide first-line therapy decisions, enabling a personalized management approach for patients with EoE.

Data availability statement: The data that support the findings of this study are available from the corresponding author on reasonable request.

**Key messages**

• Four SNPs (*ABCB1* rs1128503, *KCNJ2* rs312691, *STAT6* rs841718, and *TGFB1* rs8179181) had an effect in determining EoE phenotype.
• *CAPN14* rs74732520-CG/GG and rs77569859-TC/CC genotypes were associated with lower peak eosinophil counts at diagnosis.
• *IL13* rs1800925-TT was associated with an increased risk of asthma and conjunctivitis, whereas *ABCB1* rs2032582-TT was linked to a reduced risk of dermatitis.

## Disclosure statement

L.R.-A. is a recipient of a predoctoral contract for Health Research Training—PFIS grant (grant no. FI22/00013) from the Instituto de Salud Carlos III, Spanish Ministry of Health—Social Services and Equality, which is partly funded by the European Social Fund. P.S.-C. is financed by a predoctoral fellowship (grant no. FPI UAM-2021). This work was also supported by 2 grants from the Instituto de Salud Carlos III to A.J.L. (grant no. PI21/01036) and E.J.L.-M. (grant no. PI21/00579), cofunded by the European Union through the European Regional Development Fund (“A way of making Europe”). The funders had no role in the writing of the article or in the decision to publish the results.

Disclosure of potential conflict of interest: The authors declare that they have no relevant conflicts of interest.
