# Supplementary material for: Genetic variants associated with clinical characteristics and atopies in eosinophilic esophagitis
Source: J Allergy Clin Immunol Glob. 2026 Jun 4;5(5):100745. doi: 10.1016/j.jacig.2026.100745 (PMC13331977; doi:10.1016/j.jacig.2026.100745)
Supplement: Supplementary Tables S1-S8 [file mmc1.docx]

**Table S1.** Description of SNPs included in the Open Array platform, RefSeq nomenclature, frequencies of genotypes and alleles, and wild type allele in our cohort.

| **Gen** | **SNP** | **RefSeq** | **n (%)** | **Frequencies** | | | | | **Wild type in our cohort** |
| --- | --- | --- | --- | --- | --- | --- | --- | --- | --- |
|  |  |  |  | **Hm wild** | **Ht** | **Hm variant** | **Wild allele (E/C)** | **Variant allele (E/C)** |  |
| *ABCB1* | rs1045642 | NG_011513.1:g.208920T>C | 624 (99.4%) | 30.1% | 50.6% | 19.2% | 52.1%/55.4% | 47.9%/44.6% |  |
| *ABCB1* | rs1128503 | NG_011513.1:g.167964T>C | 624 (99.4%) | 35.4% | 48.6% | 16.0% | 57.0%/59.7% | 43.0%/40.3% | C |
| *ABCB1* | rs2032582 | NG_011513.1:g.186947T>G/A | 627 (99.8%) | 37.5% | 45.3% (GT) | 14.5% (TT) | 55.1%/60.7% | 44.8%/37.8% (T)  0.1%/1.5% (A) | G |
|  |  |  |  |  | 1.3% (GA) | 0.3% (AA) |  |  |  |
|  |  |  |  |  | 1.1% (TA) |  |  |  |  |
| *ABCG2* | rs2231142 | NG_032067.2:g.105152C>A | 627 (99.8%) | 88.2% | 11.6% | 0.2% | 89.7%/94.0% | 10.3/6.0% |  |
| *ANKRD27* | rs3815700 | NC_000019.10:g.32602346T>C^†^ | 624 (99.4%) | 76.6% | 21.5% | 1.9% | 87.4%/87.3% | 12.6%/12.7% |  |
| *ATP4A* | rs2733743 | NC_000019.10:g.35560067A>G^†^ | 623 (99.2%) | 85.2% | 14.3% | 0.5% | 89.8%/92.4% | 10.2%/7.6% | G |
| c11orf30 | rs55646091 | NC_000011.10:g.76588387G>A^†^ | 612 (97.5%) | 81.0% | 18.0% | 1.0% | 95.4%/90.0% | 4.6%/10.0% |  |
| *CAPN14* | rs76562819 | NC_000002.12:g.31217741A>G^†^ | 617 (98.2%) | 88.3% | 11.3% | 0.3% | 95.5%/94.0% | 4.5%/6.0% |  |
| *CAPN14* | rs74732520 | NC_000002.12:g.31173526C>G^†^ | 623 (99.2%) | 85.6% | 13.8% | 0.6% | 97.6%/92.5% | 2.4%/7.5% |  |
| *CAPN14* | rs77569859 | NC_000002.12:g.31188421T>C^†^ | 625 (99.5%) | 90.1% | 9.4% | 0.5% | 95.2%/94.8% | 4.8%/5.2% |  |
| *CCL26* | rs2302009 | NG_015989.1:g.25067T>G | 625 (99.5%) | 59.8% | 33.9% | 6.2% | 75.1%/76.8% | 24.9%/23.2% |  |
| chr14:20928443 | rs7141958 | NC_000014.9:g.20928443C>T^†^ | 624 (99.4%) | 82.9% | 15.9% | 1.3% | 92.6%/90.8% | 7.4%/9.2% |  |
| chr14:61613308 | rs2253681 | NC_000014.9:g.61613308A>G^†^ | 613 (97.6%) | 70.5% | 26.6% | 2.9% | 87.5%/83.8% | 12.5%/16.2% | G |
| chr18:60188738 | rs9959588 | NC_000018.10:g.60188738C>G^†^ | 622 (99.0%) | 88.3% | 11.6% | 0.2% | 95.6%/94.1% | 4.4%/5.9% |  |
| *IL13* | rs1800925 | NG_012090.1:g.3945C>T | 626 (99.7%) | 61.3% | 32.9% | 5.8% | 79.9%/77.8% | 20.1%/22.2% |  |
| *IL13* | rs20541 | NG_012090.1:g.7100A>G | 624 (99.4%) | 68.4% | 27.4% | 4.2% | 80.2%/82.1% | 19.8%/17.9% | G |
| *KCNJ2* | rs8079702 | NC_000017.11:g.70194685G>A^†^ | 487 (77.5%) | 31.2% | 48.7% | 20.1% | 56.7%/55.5% | 43.3%/44.5% | A |
| *KCNJ2* | rs312691 | NC_000017.11:g.70330197T>C^†^ | 624 (99.4%) | 51.3% | 40.9% | 7.9% | 75.4%/71.7% | 24.6%/28.3% |  |
| *SLCO1B1* | rs4149056 | NG_011745.1:g.52422T>C | 624 (99.4%) | 73.1% | 24.2% | 2.7% | 84.1%/85.2% | 15.9%/14.8% |  |
| *STAT6* | rs1059513 | NG_021272.2:g.41214A>G | 627 (99.8%) | 79.1% | 20.1% | 0.8% | 89.4%/89.2% | 10.6%/10.8% |  |
| *STAT6* | rs2598483 | NG_021272.2:g.24018G>A | 623 (99.2%) | 75.4% | 22.6% | 1.9% | 88.9%/86.8% | 11.1%/13.2% |  |
| *STAT6* | rs3024974 | NG_021272.2:g.38178C>T | 624 (99.4%) | 78.4% | 20.5% | 1.1% | 90.7%/88.6% | 9.3%/11.4% |  |
| *STAT6* | rs324011 | NG_021272.2:g.28741G>A | 627 (99.8%) | 37.3% | 48.6% | 14.0% | 61.9%/61.6% | 38.1%/38.4% |  |
| *STAT6* | rs324015 | NG_021272.2:g.40823A>G | 616 (98.1%) | 65.1% | 32.0% | 2.9% | 76.2%/81.1% | 23.8%/18.9% |  |
| *STAT6* | rs841718 | NG_021272.2:g.37927C>T | 622 (99.0%) | 38.6% | 49.0% | 12.4% | 59.5%/63.1% | 40.5%/36.9% | T |
| *TGFB1* | rs1800469 | NG_013364.1:g.4536T>C | 623 (99.0%) | 39.7% | 47.1% | 13.2% | 68.4%/63.2% | 31.5%/36.8% | C |
| *TGFB1* | rs1800470 | NG_013364.1:g.5911C>T | 616 (98.1%) | 32.0% | 50.2% | 17.9% | 52.5%/57.1% | 47.5%/42.9% | T |
| *TGFB1* | rs1800472 | NG_013364.1:g.16972C>T | 625 (99.5%) | 93.0% | 7.0% | 0% | 96.9%/96.5% | 3.1%/3.5% |  |
| *TGFB1* | rs8179181 | NG_013364.1:g.26626C>T | 623 (99.2%) | 57.1% | 37.2% | 5.6% | 76.4%/75.8% | 23.6%/24.2% |  |
| *TLR3* | rs3775292 | NG_007278.1:g.17717C>G | 623 (99.2%) | 57.0% | 37.1% | 5.9% | 80.6%/75.5% | 19.4%/24.5% | G |
| *TSLP* | rs3806932 | NC_000005.10:g.111069977A>G^†^ | 617 (98.2%) | 47.0% | 43.9% | 9.1% | 55.3%/69.0% | 44.7%/31.0% |  |
| *TSLP* | rs10062929 | NC_000005.10:g.111072481C>A^†^ | 624 (99.4%) | 83.7% | 16.0% | 0.3% | 86.0%/91.7% | 14.0%/8.3% |  |
| *TSLP* | rs1898671 | NC_000005.10:g.111072304C>T^†^ | 625 (99.5%) | 25.4% | 51.7% | 22.9% | 65.7%/51.3% | 34.3%/48.7% |  |
| *TSLP* | rs2289276 | NC_000005.10:g.111071809C>T^†^ | 626 (99.7%) | 61.3% | 33.4% | 5.3% | 70.4%/78.0% | 29.6%/22.0% |  |
| *TSLP/*  *WDR36* | rs1438673 | NG_008979.1:g.44630C>T | 599 (95.4%) | 41.1% | 47.1% | 11.9% | 49.7%/64.6% | 50.3%/35.4% |  |
| *WDR36* | rs7723819 | NG_008979.1:g.4478G>A | 625 (99.5%) | 46.2% | 44.6% | 9.1% | 54.5%/68.6% | 45.5%/31.4% |  |
| *TBL1Y*^‡^ | rs768983 | NC_000024.10:g.6950250C>T^†^ | 460 (73.4%) | 99.8% | 0.2% | 0% | 96.3%/99.9% | 3.7%/0.1% |  |

The last column indicates when the most frequent allele in our cohort was different from the reference allele in RefSeq. C=in our cohort; E=in European population according to databases; hm=homozygous; ht: heterozygous. ^†^No information in RefSeq and the data provided correspond to GRCh38 annotation. ^‡^*TBL1Y* was used as a control for male sex.

**Table S2.** Consequences of the SNPs included in the analysis according to dbSNP and Ensembl (when annotations differed, both are shown as follows: dbSNP: gene consequence/Ensembl: most severe consequence) and functional effect according to ClinVar if reported.

| **Gen** | **SNP** | **Consequence** | **Functional/Clinical effect** |
| --- | --- | --- | --- |
| *ABCB1* | rs1045642 | Missense variant | Benign |
|  | rs1128503 | Synonymous variant | Benign/Drug response |
|  | rs2032582 | Missense variant | Benign/Drug response/Risk factor IBD |
| *ABCG2* | rs2231142 | Stop gained variant | Benign/Drug response |
| *ANKRD27* | rs3815700 | Intron variant | - |
| *ATP4A* | rs2733743 | Missense variant | Benign |
| c11orf30 | rs55646091 | Regulatory region variant | - |
| *CAPN14* | rs76562819 | Intron variant | - |
|  | rs74732520 | 3’ UTR variant | - |
|  | rs77569859 | Intron variant | - |
| *CCL26* | rs2302009 | 3’ UTR variant | - |
| chr14:20928443 | rs7141958 | Intron variant | - |
| chr14:61613308 | rs2253681 | Intron variant | - |
| chr18:60188738 | rs9959588 | Intergenic variant | - |
| *IL13* | rs1800925 | Intron variant / Intergenic variant | Risk factor (asthma) |
|  | rs20541 | Missense variant | Benign/Risk factor (asthma, allergic rhinitis) |
| *KCNJ2* | rs8079702 | Intergenic variant | - |
|  | rs312691 | Intron variant | - |
| *SLCO1B1* | rs4149056 | Missense Variant | Benign/Drug response |
| *STAT6* | rs1059513 | Non coding transcript variant/3’ UTR variant | - |
|  | rs2598483 | 2KB Upstream variant/Regulatory region variant | - |
|  | rs3024974 | Intron variant | - |
|  | rs324011 | Intron variant/5’ UTR variant | - |
|  | rs324015 | Non coding transcript variant/3’ Prime UTR variant | - |
|  | rs841718 | Intron variant | - |
| *TGFB1* | rs1800469 | 2KB Upstream variant / Intron variant | Benign |
|  | rs1800470 | Missense variant | Benign/Risk factor (cystic fibrosis, breast cancer) |
|  | rs1800472 | Missense variant | Benign |
|  | rs8179181 | Intron variant | Benign |
| *TLR3* | rs3775292 | Intron variant / 5’ UTR variant | Benign |
| *TSLP* | rs3806932 | 2KB Upstream variant/Regulatory region variant | - |
|  | rs10062929 | Intron variant | - |
|  | rs1898671 | Intron variant | - |
|  | rs2289276 | Intron variant/5’ UTR variant | - |
| *TSLP/WDR36* | rs1438673 | Intergenic variant | - |
| *WDR36* | rs7723819 | 2KB Upstream variant / Intron variant | - |
| *TBL1Y*^‡^ | rs768983 | Intron variant | - |

**Table S3.** Ancestry and country of birth of the patients included in our cohort.

| **Ancestry, n (%)** | | **Country** | **n (%)** |
| --- | --- | --- | --- |
| European, 618 (98.4) | Iberian, 615 (99.5) | Spain | 613 (97.6) |
|  |  | Portugal | 2 (0.3) |
|  | Other, 3 (0.5) | France | 1 (0.2) |
|  |  | Germany | 1 (0.2) |
|  |  | Romania | 1 (0.2) |
| Latin American / Admixed, 7 (1.1) | | Venezuela | 4 (0.6) |
|  |  | Colombia | 2 (0.3) |
|  |  | Argentina | 1 (0.2) |
| North African, 3 (0.5) | | Morocco | 3 (0.5) |

**Table S4.** Characteristics of the patients after dividing into discovery and validation cohorts.

|  |  | **Discovery** | **Validation** |
| --- | --- | --- | --- |
| Type of data, n (%) | Retrospective | 374 (85.2) | 165 (87.3) |
|  | Prospective | 65 (14.8) | 24 (12.7) |
| Male sex, n (%) | | 330 (75.2) | 139 (73.5) |
| Age (mean±SD) | | 31.7±15.2 | 34.1±15.1 |
| EREFS | Mean±SD | 3.20±1.63 | 3.01±1.68 |
|  | Median (IQR) | 3 (2) | 3 (2) |
| Peak of eos/hpf | Mean±SD | 64.5±47.1 | 58.9±41.9 |
|  | Median (IQR) | 51 (47) | 49 (41) |
| Phenotype, n (%) | Inflammatory | 359 (81.8) | 154 (81.5) |
|  | Mixed/ Stricturing | 79 (18.0) | 35 (18.5) |
|  | Unknown | 1 (0.2) | - |
| Rhinitis, n (%) | Presence | 244 (55.6) | 104 (55.0) |
| Conjunctivitis, n (%) | Presence | 173 (39.4) | 69 (36.5) |
| Asthma, n (%) | Presence | 159 (36.2) | 66 (34.9) |
| Dermatitis, n (%) | Presence | 73 (16.6) | 36 (19.0) |
| Food allergy, n (%) | Presence | 136 (31.0) | 41 (21.7) |
| At least one atopy, n (%) | | 326 (74.3) | 136 (72.0) |
| Smoker or former smoker, n (%) | | 86 (19.6) | 30 (15.9) |

**Table S5.** SNPs previously associated with eosinophilic esophagitis (EoE) which did not show a different proportion between our patients with EoE and the general European population.

| **Gene** | **SNP** | **Most frequent allele (%)** | | **Less frequent allele (%)** | | **p-value compared with EP** | **References** |
| --- | --- | --- | --- | --- | --- | --- | --- |
|  |  | **EP** | **C** | **EP** | **C** |  |  |
| *ANKRD27* | rs3815700 | 87.4 | 87.3 | 12.6 | 12.7 | 0.95 | Sleiman, 2014 |
| *CAPN14* | rs76562819 | 95.5 | 94.0 | 4.5 | 6.0 | 0.09 | Kottyan, 2014 |
| *CAPN14* | rs77569958 | 95.2 | 94.8 | 4.8 | 5.2 | 0.59 | Kottyan, 2014 |
| *CCL26* | rs2302009 | 75.1 | 76.8 | 24.9 | 23.2 | 0.31 | Blanchard, 2006 |
| *STAT6* | rs324011^†^ | 61.9 | 61.6 | 38.1 | 38.4 | 0.89 | Rothenberg, 2010 |

C: cohort of our study; EP: European population. ^†^In linkage disequilibrium with rs167769 and rs12368672 in European population (both p<0.001).

**Table S6.** Patients with mixed/stricturing phenotype (m/s) for the qualitative variables included in the multivariate model. The percentage of patients in each group of the included variable is described in the second column.

|  | | **n (%) of m/s phenotype** | **% in cohort** |
| --- | --- | --- | --- |
| Age group at diagnosis | Adult (≥18 years old) | 99 (20.0) | 78.9 |
|  | Child (<18 years old) | 15 (11.4) | 21.1 |
| Sex | Male | 97 (20.9) | 74.0 |
|  | Female | 17 (10.4) | 26.0 |
| Food allergies | Yes | 46 (26.1) | 28.2 |
|  | No | 68 (15.1) | 71.8 |
| Smoking | Yes or former smoker | 16 (13.8) | 19.0 |
|  | No | 97 (19.6) | 81.0 |
| *ABCB1* rs1128503 | CC | 37 (16.7) | 35.4 |
|  | TC/TT | 77 (19.2) | 64.6 |
| *KCNJ2* rs312691 | TT/TC | 107 (18.6) | 92.1 |
|  | CC | 5 (10.2) | 7.9 |
| *STAT6* rs841718 | TT | 50 (20.8) | 38.6 |
|  | TC/CC | 60 (15.7) | 61.4 |
| *TGFB1* rs8179181 | CC/CT | 104 (17.7) | 94.4 |
|  | TT | 8 (22.9) | 5.6 |

**Table S7.** SNPs that showed a significant change for the EREFS score and peak of eosinophils/hpf in the discovery cohort but not in the validation cohort.

| **Variable** | **Gene and SNP** | **Genotype** | **n (%)** | **Mean±SD** | **p-value DC** | **p-value VC** |
| --- | --- | --- | --- | --- | --- | --- |
| EREFS score | *SLCO1B1* rs4149056 | TT | 295 (74.1) | 3.36±1.71 | 0.001 | 0.419 |
|  |  | TC, CC | 103 (25.9) | 2.78±1.29 |  |  |
|  | *TLR3* rs3775292 | GG, GC | 372 (93.5) | 3.16±1.61 | 0.034 | 0.964 |
|  |  | CC | 26 (6.5) | 3.92±1.79 |  |  |
| Peak of eosinophils/hpf | *ABCB1* rs1128503 | CC | 147 (38.4) | 58.7±41.2 | 0.032 | 0.420 |
|  |  | CT, TT | 236 (61.6) | 68.2±50.3 |  |  |
|  | *ABCB1* rs2032582 | GG | 154 (40.2) | 59.3±41.4 | 0.040 | 0.392 |
|  |  | GT, TT | 229 (59.8) | 68.2±50.4 |  |  |
|  | *CAPN14* rs76562819 | AA | 339 (88.7) | 66.9±48.9 | 0.005 | 0.201 |
|  |  | AG, GG | 43 (11.3) | 47.0±24.4 |  |  |
|  | *TGFB1* rs1800472 | CC | 352 (92.1) | 62.0±39.7 | 0.015 | 0.245 |
|  |  | CT | 30 (7.9) | 95.4±95.9 |  |  |

DC: discovery cohort; EREFS: edema, rings, exudates, furrows, and strictures; hfp: high power field; SD: standard deviation; SNP: single nucleotide polymorphism; VC: validation cohort.

**Table S8.** SNPs that showed a significant change for any atopic condition in the discovery cohort but not in the validation cohort.

| **Variable** | **Gene and SNP** | **Genotype** | **n (%)** | **Presence of AC** | **p-value DC** | **p-value VC** |
| --- | --- | --- | --- | --- | --- | --- |
| Asthma | *STAT6* rs3024974 | CC | 340 (80.0) | 39.4% | 0.009 | 0.794 |
|  |  | CT, TT | 100 (22.0) | 25.0% |  |  |
| Dermatitis | *ABCB1* rs1128503 | CC, CT | 365 (83.5) | 18.9% | 0.006 | 0.470 |
|  |  | TT | 72 (16.5) | 5.6% |  |  |
|  | c11orf30 rs55646091 | GG | 351 (81.8) | 18.5% | 0.041 | 0.586 |
|  |  | GA, AA | 78 (18.2) | 9.0% |  |  |
|  | *IL13* rs20541 | GG, GA | 420 (96.1) | 16.0% | 0.036 | 0.526 |
|  |  | AA | 17 (3.9) | 35.3% |  |  |
|  | *TLR3* rs3775292 | GG, GC | 409 (93.8) | 17.8% | 0.016 | 0.347 |
|  |  | CC | 27 (6.2) | 0 % |  |  |

AC: atopic condition; DC: discovery cohort; SNP: single nucleotide polymorphism; VC: validation cohort.
